# Mechanisms of Waterlogging Tolerance in Plants: Research Progress and Prospects

**DOI:** 10.3389/fpls.2020.627331

**Published:** 2021-02-10

**Authors:** Jiawei Pan, Rahat Sharif, Xuewen Xu, Xuehao Chen

**Affiliations:** ^1^School of Horticulture and Plant Protection, Yangzhou University, Yangzhou, China; ^2^Joint International Research Laboratory of Agriculture and Agri-Product Safety, Yangzhou University, Yangzhou, China

**Keywords:** waterlogging stress, morphological structure, photosynthesis, energy metabolism, plant hormones, molecular mechanism

## Abstract

Waterlogging is one of the main abiotic stresses suffered by plants. Inhibition of aerobic respiration during waterlogging limits energy metabolism and restricts growth and a wide range of developmental processes, from seed germination to vegetative growth and further reproductive growth. Plants respond to waterlogging stress by regulating their morphological structure, energy metabolism, endogenous hormone biosynthesis, and signaling processes. In this updated review, we systematically summarize the changes in morphological structure, photosynthesis, respiration, reactive oxygen species damage, plant hormone synthesis, and signaling cascades after plants were subjected to waterlogging stress. Finally, we propose future challenges and research directions in this field.

## Introduction

Plants achieve normal growth through the coordination of water absorption by the roots with transpiration from the leaves. Sufficient water is a prerequisite for normal growth of plants, but saturation of the soil water-holding capacity, or even super-saturation, easily leads to waterlogging stress. The inhibition of root respiration and accumulation of toxic substances during waterlogging stress have adverse effects not only on vegetative growth, but also on reproductive growth, eventually leading to yield loss or even complete harvest failure (Hirabayashi et al., 2013; Xu et al., 2014; Herzog et al., 2016; Tian et al., 2019; Ding et al., 2020; Zhou et al., 2020). Therefore, in the context of global warming, with predictions of more frequent and/or heavy rainfall and frequent flood disasters, there is a pressing need to study plant waterlogging tolerance and its mechanisms in order to maintain successful agriculture and promote effective adaptations to the changing climate (Bailey-Serres et al., 2012; Nishiuchi et al., 2012; Mondal et al., 2020).

During waterlogging, leaf stomata close, whereas chlorophyll degradation, leaf senescence, and yellowing reduce the ability of leaves to capture light and ultimately lead to a decline in photosynthetic rate (Kuai et al., 2014; Yan et al., 2018). Waterlogging removes air from soil pores, resulting in blocked gas exchange between soil and atmosphere; at the same time, the oxygen diffusion rate in water is only 1/10,000 of that in air. Consequently, oxygen availability in waterlogged soil is greatly restricted, resulting in suppressed roots respiration, decreased root activity, and energy shortage (van Veen et al., 2014). Plants can temporarily maintain energy production to some extent during hypoxia caused by waterlogging, *via* glycolysis and ethanol fermentation. However, prolonged duration of waterlogging and anaerobic respiration ultimately leads to the accumulation of toxic metabolites such as lactic acid, ethanol, and aldehydes, combined with an increases in reactive oxygen species (ROS), notably hydrogen peroxide, thus eventually leading to cell death and plant senescence (Xu et al., 2014; Zhang P. et al., 2017). Hindered gaseous exchange can also lead to rapid accumulation or degradation of plant hormones and further affect plant waterlogging tolerance (Hattori et al., 2009; Kuroha et al., 2018). Although most plants perform poorly when waterlogged, they can adapt to the damage caused by such environmental stress through various strategies (Fukao et al., 2006; Xu et al., 2016; Doupis et al., 2017; Yin et al., 2019).

The flooding stress, which further causes the submergence; hypoxia; and waterlogging stress are the main limiting factors of crop productivity. Flooding imposes submergence and ultimately raises the ground water table, which creates a hypoxic condition in the rhizosphere. The hypoxic condition in the rhizosphere restricts the oxygen uptake by causing an anaerobic environment, which further leads to plant death (Fukao et al., 2019), Therefore, the flooding, submergence, and waterlogging stress are interconnected and affect the plant in nearly similar fashion (Fukao et al., 2019).

**FIGURE 1:**
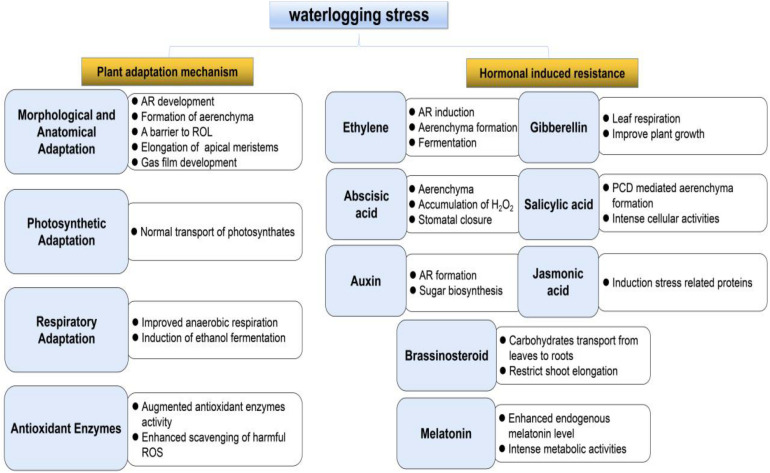
Schematic representation of plant response to waterlogging stress and hormonal effects resistance in plants.

In this updated review, we summarize the progress of research on plant adaptations to waterlogging stress with a focus on six aspects: morphological and anatomical adaptations, photosynthesis, respiration, ROS injury, plant hormone biosynthesis and signaling cascades, and genetic engineering in enhancing tolerance of plant against waterlogging stress (Figure 1). Finally, the future challenges and research direction in this field are discussed, aiming to provide a source of reference and recommendations for further research on plant waterlogging resistance.

## Morphological and Anatomical Adaptation

Most plants are sensitive to waterlogging, as the diffusion rates of O_2_ and CO_2_ in roots and stems of plants decrease significantly during waterlogging, and photosynthesis and respiration are significantly inhibited. However, various morphological changes occur in some plants and can relieve root respiratory depression and damage caused by disrupted energy metabolism under waterlogging. Morphological changes are mainly manifested as the formation of adventitious roots (ARs) or other aeration tissues, rapid elongation of apical meristematic tissue, barriers to radial oxygen loss (ROL), and the formation of air films in the upper cuticle (Hattori et al., 2009; Pedersen et al., 2009; Yamauchi et al., 2017; Qi et al., 2019).

Formation of ARs is a typical adaptive change in morphology (Steffens and Rasmussen, 2016). During extended waterlogging, ARs develop in the internodes on the hypocotyl or at the base of the stem, where they promote the exchange of gases and the absorption of water and nutrients. To a certain extent, AR formation can replace the primary roots that die because of hypoxia stress, maintaining metabolic cycles, and enabling normal growth and development (Xu et al., 2016; Eysholdt−Derzsó and Sauter, 2019). The newly formed ARs contain more aerenchyma than the primary roots, which augment both O_2_ uptake and diffusion ability (Visser and Voesenek, 2005).

Programmed cell death and degradation occur in cortical cells of plant root under hypoxia, producing tissue cavities and leading to aerenchyma formation. Aerenchyma not only can transport O_2_ from non-waterlogged tissue to the root system, but also discharge CO_2_ and toxic volatile substances from waterlogged tissue. Therefore, aerenchyma provides the possibility of gas exchange within plants and is vital for maintaining the normal physiological metabolism in the cells of waterlogged roots (Drew et al., 2000; Evans, 2004; Yamauchi et al., 2013).

Radial oxygen loss refers to the fact that O_2_ can be consumed by respiration during the longitudinal transport of O_2_ along the aerenchyma to the root tip and can also be lost by lateral leakage into the intercellular spaces of the rhizosphere (Yamauchi et al., 2018). Plants are able to produce a barrier to ROL, thereby reducing the loss of O_2_ to the intercellular spaces of the rhizosphere and O_2_ diversion between and around the root tip (Pedersen et al., 2020). Abiko and Miyasaka (2020) used methylene blue to stain the ARs of taro [*Colocasia esculenta* (L.) Schott] after 8 days of waterlogging and found that the root tips turned blue, whereas no blue areas appeared in the middle sections of the roots. This indicated that O_2_ leakage was detected only near the root tip along the intercellular spaces of the rhizosphere, as the ROL barrier was formed in the middle of the root and prevented lateral losses (Shiono et al., 2011). The formation of the ROL barrier inhibited the release of O_2_ in the primordia of aerenchyma in rice (*Oryza sativa*) after 12 h of waterlogging (Shiono et al., 2011). Moreover, deepwater rice cultivars form a tighter ROL barrier under low oxygen conditions than upland rice (Colmer, 2002).

The rapid elongation of plant apical meristems is another adaptation of plants to waterlogging. The rapid elongation of tender stems and internodes facilitates escape from the anoxic environment and contact with the air as soon as possible, thereby enabling normal respiration (Kuroha et al., 2018). This response is known as low oxygen escape syndrome (LOES). Internodes of deepwater rice cultivars elongate rapidly: waterlogging induces accumulation of ethylene (ET) and promotes synthesis of gibberellins (GAs) (largely GA_4_), thus promoting internode elongation (Kuroha et al., 2018) (see also Waterlogging stress mediated by plant hormones).

As an adaptation to waterlogging, some plants maintain a gas film on the leaf surface when submerged (Winkel et al., 2016; Kurokawa et al., 2018). The gas film promotes the entry of O_2_ in darkness and CO_2_ when in light, thus contributing to the maintenance of aerobic respiration and photosynthesis. After artificial removal of the gas film in waterlogged rice, the underwater net photosynthetic rate was found to be only 20% of that with the gas film in place (Pedersen et al., 2009).

## Photosynthetic Adaptation

During waterlogging, stomatal conductance of leaves decreases, stomatal resistance increases, stomatal closure increases, and absorption of CO_2_ is reduced (Li et al., 2010). However, plants need CO_2_ and light for photosynthesis to maintain growth and development. Under prolonged waterlogging condition, the enzyme activities related to photosynthesis were inhibited; the chlorophyll synthesis ability of leaves decreased, leading to leaf senescence, yellowing, and peeling; the formation of new leaves was blocked, and then the photosynthetic rate decreased, finally leading to death of the plants (Voesenek et al., 2006;Wu and Yang, 2016).

Photosynthetic pigments are the material basis of plant photosynthesis, and the change of pigment content and composition directly affects the photosynthetic rate. Anee et al. (2019) conducted waterlogging experiments on sesame (*Sesamum indicum* L.) seeds for 2, 4, 6, and 8 days to explore the changes in physiological and biochemical characteristics with time under waterlogging. The content of the photosynthetic pigments, chlorophyll A, B chlorophyll A + B, and carotenoids, was significantly lower in waterlogged seeds than in the unwaterlogged control; as the content of photosynthetic pigments decreased, the photosynthetic capacity also decreased.

The enzyme rubisco catalyzes the first step of both the photosynthetic carbon cycle and photorespiration and plays a key role in regulating the photosynthetic rate. After 24 h upon waterlogging stress, the expression of rubisco and rubisco activase genes in cotton (*Gossypium hirsutum* L.) leaves was down-regulated; a reduction in net photosynthetic rate of cotton was mainly caused by lower rubisco activity. Sucrose and starch are the main products of photosynthesis in most plants. Sucrose is the main transport carbohydrate from source to sink, a process that is very sensitive to waterlogging. The decreased photosynthetic rate, sucrose conversion rate, and initial rubisco activity directly reduced the boll weight of waterlogged cotton. The enzyme sucrose synthase is central to the metabolic breakdown of sucrose required for cellulose biosynthesis; increased gene expression and enzyme activity of sucrose synthase during waterlogging were associated with prolonging the period of rapid accumulation of seed fiber weight, tending to reduce the phenomenon of boll weight decline caused by waterlogging (Kuai et al., 2014).

## Respiratory Adaptation

The generation of energy is crucial for plant growth and development. The lack of energy caused by hypoxia and consequent inhibition of root respiration are some of the most serious problems faced by plants under waterlogging (Loreti et al., 2016). In cultivated soil, the concentration of dissolved oxygen in water is generally approximately 0.23 mol/m^3^, whereas in waterlogged conditions, the concentration of dissolved oxygen in water is less than 0.05 mmol/m^3^. The diffusion rate of O_2_ in waterlogged soil is only 1/10,000 of that in the air. O_2_ is the electron acceptor at the end of the mitochondrial electron transport chain. Decreased O_2_ availability rapidly inhibits the production of adenosine triphosphate (ATP) by interfering with the electron transport chain, leading to inhibition of mitochondrial respiration (Bailey-Serres and Voesenek, 2008; Limami et al., 2014). Plants need to obtain the necessary energy supply through glycolysis and ethanol fermentation so as to cope with the energy shortage caused by waterlogging stress (Baxter-Burrell et al., 2002). However, 1 mol glucose can produce 36 to 38 mol ATP through the tricarboxylic acid cycle, whereas only 2 mol ATP can be obtained through glycolysis and ethanol fermentation. Therefore, plants need to accelerate glycolysis and ethanol fermentation in order to obtain the necessary amounts of ATP needed to sustain life.

Pyruvate accumulated from glycolysis can be used for anaerobic fermentation. Pyruvate fermentation produces energy in two different ways, producing lactic acid either *via* lactate dehydrogenase (LDH) or *via* pyruvate decarboxylase (PDC) turning pyruvate into acetaldehyde, which is then reduced to ethanol by alcohol dehydrogenase (ADH) (Zabalza et al., 2009; Caruso et al., 2012; Borella et al., 2019). ADH and PDC play key roles in the ethanol fermentation pathway, and their activity is usually considered as one of the important indexes reflecting the tolerance of plants to waterlogging. Waterlogging-tolerant plants can improve the ethanol fermentation rate by regulating the expression of *ADH*, *PDC*, and other related enzyme genes, which can temporarily provide energy for the growth of plants under waterlogging (Zhang P. et al., 2017). Therefore, fermentation is a necessary process of energy metabolism under waterlogging, as shown by the up-regulated expression of anaerobic metabolism genes such as *PDCs* and *ADHs* in cucumber, cotton, and soybean (Komatsu et al., 2011; Xu et al., 2014; Zhang et al., 2015). The seed germination ability of *GmADH2*-transgenic soybeans was enhanced under waterlogging, and the *GmADH2* gene was induced during glycolysis and ethanol fermentation (Tougou et al., 2012). The overexpression of kiwifruit *PDC1* gene in transgenic *Arabidopsis* enhanced waterlogging tolerance (Zhang J.Y. et al., 2016). These results indicate that *PDC* and *ADH* genes play key roles in plant waterlogging tolerance.

Lactate dehydrogenase also participates in the waterlogging stress response, alongside PDC and ADH. Overexpression of *LDH* significantly enhanced the PDC activity and hypoxia resistance of *Arabidopsis*, whereas LDH loss of function mutant *ldh* showed the opposite phenotype (Dolferus et al., 2008). Therefore, lactic acid fermentation is an important pathway in response to waterlogging stress in some plants. The transcript abundances of the ethanol dehydrogenase genes *ADH1-1*, *ADH1-2*, *ADH1-3*, and PDC genes *PDC1* and *PDC2* were down-regulated in *Petunia* plants in which an ET-responsive element–binding factor *PhERF2* was silenced, whereas they were up-regulated in *PhERF2-*overexpressing plants. In contrast, the expression of LDH gene *LDH* was up-regulated in *PhERF2-*silenced lines and down-regulated in *PhERF2-*overexpressing lines. This result suggests that the main pathway for NAD^+^ regeneration in *PhERF-*overexpressing plants is ethanol fermentation, whereas *PhERF2-*silenced plants might rely on lactic acid fermentation in response to waterlogging stress (Yin et al., 2019).

Although the energy generated *via* glycolysis and ethanol fermentation can temporarily alleviate the energy deficiency caused by the inhibition of respiration in roots, the accumulation of toxic substances such as lactic acid, alcohols, aldehydes, and other anaerobic metabolites eventually leads to plant death as the time of waterlogging is prolonged (Tamang et al., 2014).

## Damage by Reactive Oxygen Species

Reactive oxygen species are a normal product of plant cell metabolism. Insufficient O_2_ will also lead to increases in intracellular ROS under waterlogging stress (Bailey-Serres and Chang, 2005; Pucciariello et al., 2012). For example, superoxide radicals (⋅O_2_), hydroxyl radicals (⋅OH), and hydrogen peroxide (H_2_O_2_) have strong oxidizing activity that can lead to lipid peroxidation and delipidation of leaf membranes, oxidative damage to proteins, oxidative damage to DNA, and severe damage to cell membranes and organelles (Sharma et al., 2012; Baxter et al., 2014).

Although excessive ROS are harmful to plant cells, ROS can also act as signaling molecules in plant cells under stress. Plant NADPH oxidase is a key enzyme in the production of ROS and plays a vital role in ROS-mediated signal transduction. The expression of NADPH oxidase–related gene *Atrboh D*, a gene associated with ROS production, is induced by waterlogging and positively regulates the production of H_2_O_2_ and the increase of *ADH1* gene expression in *Arabidopsis*. Therefore, this signal improves the capacity for ethanol fermentation and increases the survival rate of plants under waterlogging (Sun et al., 2018). Analysis of the *atrboh d* mutant by Yang and Hong (2015) showed that *AtRboh D* is involved in the primary hypoxia signaling pathway and can regulate the transcription of ET synthesis gene *ACC synthetase7/8 (ACS7/8*), as well as the regulation of hypoxia-induced downstream genes such as *ERF73/HRE*1 and *ADH1* and the expression of genes encoding peroxidase and cytoplasmic P450. Subsequently, Liu et al. (2017) analyzed the single mutant *atrboh d* and *atrboh f* and the double-mutant *atrbohd/f.* Both *Atrboh D* and *Atrboh F* play a role in hypoxia signal through the production of ROS, promoting the increase of Ca^2+^ and mediating hypoxia-induced expression of downstream genes, such as *ADH1*, *PDC1*, *ERF73*, *MYB2*, *LDH*, *SUS1*, *SUS4*, *HsfA2*, and *HSP18.2*, thus improving the tolerance of *Arabidopsis* to hypoxia stress. These findings provide new insights into the adaptation mechanism of *Rboh* gene regulation under waterlogging stress in plants.

H_2_O_2_ is an essential signaling molecule involved in ET-induced epidermal cell death. The formation of aerenchyma in rice stems is controlled by H_2_O_2_, indicating that ROS play a key role in regulating various cell death processes in rice (Steffens et al., 2011). H_2_O_2_ plays a role in primary hypoxia signaling by regulating ET signal transduction and modulating the transcription of downstream hypoxia-induced genes such as *ERF73/HRE1* and *ADH1* in *Arabidopsis* (Yang, 2014). This signal promoted the capacity for ethanol fermentation, temporarily alleviated the energy shortage, and improved the adaptability of the plants to waterlogging.

Under waterlogging stress, plants can rely on antioxidant enzyme systems and other active antioxidants to maintain the dynamic balance of ROS, thus reducing the extent of oxidative damage (Zhang et al., 2007; Bin et al., 2010; Doupis et al., 2017; Hasanuzzaman et al., 2020). Waterlogging treatment resulted in increased activities of catalase (CAT), ascorbate peroxidase (APX), and superoxide dismutase (SOD), as well as polyphenol oxidase. Furthermore, the enzyme activity of waterlogging-resistant lines was significantly higher than that of waterlogging-sensitive lines (Bansal and Srivastava, 2012). Li (2007) took two cucumber varieties with significantly different waterlogging tolerance as test materials and found that the activities of SOD, POD, and CAT, as well as chlorophyll content, soluble sugar content, and CAT content of waterlogging-sensitive lines, decreased rapidly; there was no significant difference between waterlogging-resistant lines in the early stress (1–3 days) treatment and the control. After 3 days, they all decreased rapidly, but the extent of the decrease was smaller than that of waterlogging-sensitive lines. Several genotypes of maize were subjected to waterlogging stress. Genotypes withstanding the waterlogging stress displayed higher SOD, POD, and CAT activities (Li et al., 2018). Similarly, induced SOD and CAT activities were observed in the *Sorghum bicolor* waterlogging-resistant lines JN01 and JZ31 (Zhang R. et al., 2019). Waterlogging stress was given to barley-tolerant and -sensitive genotypes for 21 days to evaluate the antioxidant response (Luan et al., 2018). The study revealed that SOD, POD, and CAT activities were increased in both the tolerant and sensitive genotypes (Luan et al., 2018). It could be presumed that enhanced antioxidant activities under waterlogging stress can increase the tolerance of plant for a certain amount of time. However, extended waterlogging stress leads to the dysfunctioning of mitochondria, which is the key regulator of antioxidant enzyme activities (Sharif et al., 2018).

The activity of APX in eggplant roots under waterlogging was higher than that of other antioxidant enzymes, and the activity of APX was higher than that of tomato. Consequently, eggplant had higher adaptation ability to waterlogging (Lin et al., 2004). Lee et al. (2014) conducted waterlogging experiments on rapeseed seedlings and found that a CAT-encoding gene was down-regulated, whereas SOD and POD genes were up-regulated. CAT might be involved in controlling H_2_O_2_ content by converting H_2_O_2_ into O_2_. The down-regulation of this gene would then increase the content of H_2_O_2_ in the leaves of rape seedlings and eventually damage the photosynthetic organs, leading to premature aging.

The application of exogenous regulatory substances is one of the main ways to improve the antioxidant capacity of waterlogged crops (An et al., 2016). For example, application of γ-aminobutyric acid can increase the photosynthetic rate and chlorophyll content by triggering the activity of antioxidant enzymes (SOD, POD, CAT, GR, APX), suppress the malondialdehyde (MDA) contents and H_2_O_2_, and thus improve the waterlogging tolerance of maize (*Zea mays* L.) (Salah et al., 2019). The H_2_O_2_ application at low concentrations can also induce plant tolerance to stress (Hossain et al., 2015). In line with that, Andrade et al. (2018) pretreated soybean seeds with 70 mM H_2_O_2_ solution for 24 h and then subjected the seedlings to waterlogging for 32 days. The obtained results revealed that H_2_O_2_ pretreatment promoted the antioxidant system activity and net photosynthetic rate under waterlogging and at the same time reduced the production of ROS and the degree of cell membrane damage, conferring enhanced waterlogging tolerance of soybean. The above results indicate that the ROS-scavenging ability of plants can be enhanced by increasing in active antioxidant substances, and the waterlogging-resistant lines maintained high antioxidant enzyme activities that enabled them to resist oxidative damage caused by waterlogging.

## Waterlogging Stress Mediated by Plant Hormones

**FIGURE 2:**
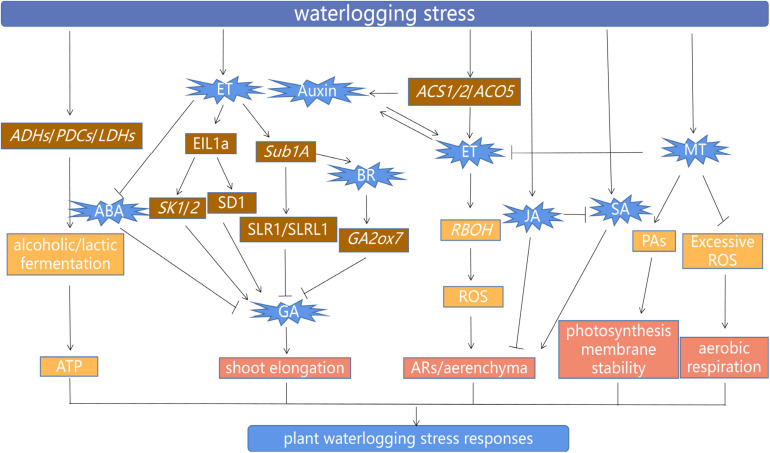
Model of waterlogging response mechanism in plants. Arrows indicate positive stimuli; lines with blocked ends denote inhibitory effects. Waterlogging-tolerant plants can improve the rate of ethanol and lactic fermentation by enhancing the expression of *ADHs*, *PDCs*, and *LDHs*, whose gene products temporarily provide ATP for the growth of plants under waterlogging. *OsEIL1a* (an ethylene-responsive transcription factor) promotes *SK1/2* transcription and also directly binds the promoter of GA biosynthesis gene *SD1*, thereby increasing the synthesis of GA, which stimulates shoot elongation. Increased ethylene levels (ET) inhibit ABA biosynthesis, which leads to increased GA content and induces shoot elongation in deepwater rice cultivars. However, in non-deepwater cultivars, *Sub1A* negatively regulates the GA response by limiting the degradation of the GA signal inhibitor protein SLR1/SLRL1, which inhibits shoot elongation. An increased BR level in Sub1A rice genotype induces the expression of GA catabolism gene *GA2ox7*, which represses GA signaling, so then shoot elongation is inhibited. In addition, the expression of *ACS* and *ACO5* is induced in rice root aerenchyma under waterlogging, thereby promoting ethylene synthesis. Ethylene accumulation enhances auxin biosynthesis and transport, and *vice versa*. Ethylene enhances the expression of RBOH (NADPH oxidase, respiratory burst oxidase homolog) and induces ROS signals, which finally lead to the formation of ARs and aerenchyma. Furthermore, SA triggers a programmed cell death response, which leads to the development of aerenchyma cells. At the same time, the accumulation of SA stimulates the formation of AR primordia. JA inhibits AR growth and inhibits the action of SA under waterlogging. The application of MT enhanced tolerance to waterlogging stress by triggering the generation of PA biosynthesis, suppression of excessive ROS, and then improved photosynthetic machinery and aerobic respiration. Interestingly, the MT-treated plants under waterlogging stress exhibited decrease expression pattern of ethylene biosynthesis and signaling gene.

Endogenous plant hormones are closely involved in the regulation of the entire life process of plants, and the balance of various hormones is the basis to ensure normal physiological metabolism, growth, and development of plants (Bartoli et al., 2013; Miransari and Smith, 2014; Wang X. et al., 2020). The plant changes the balance of synthesis and transport of plant hormones and regulates the response to waterlogging *via* complex signaling. Plant hormones, as important endogenous signals, play a central role in the mechanism of waterlogging tolerance (Benschop et al., 2006; Wu et al., 2019; Yamauchi et al., 2020). Some selected recent studies on phytohormones and plant growth regulator–mediated waterlogging tolerance in plants are presented in Table 1. A model drawing together the interactions of the various signals, growth regulators, genes, and processes involved in the response of plants to waterlogging is summarized in Figure 2.

**TABLE 1:** Recent studies on phytohormones and plant growth regulator mediated waterlogging tolerance in plants.

Plant/crop species	Exogenous phytohormones or plant growth regulator concentration applied	Experimental conditions	Functional response	References
*Solanum lycopersicum*	500 μM AVG	Four-week-old plants were flooded, and the aerial part was sprayed with 500 μM AVG 12 h before the start of flooding	AVG inhibited the AR formation	Vidoz et al., 2010
*Cucumis sativus*	1 mg/L 1-MCP or 10 μM ACC	13-day-old seedlings were pretreated with 1-MCP for 24 h; 14-day-old seedlings were subjected to waterlogging up to 2 cm above the surface of the substrate	Enhanced AR formation and recovered plant growth under waterlogging	Qi et al., 2019
*Glycine max*	50, 100, or 200 μM ethephon	During the V2 stage and the water level was maintained at 10–15 cm above the soil surface for 10 days	Induced the occurrence of ARs and increased the root surface area	Kim et al., 2018
*G. max*	1 μM ABA	Primary leaves had fully expanded (10–11 days after sowing), and the level of water or ABA solution was maintained at 3 cm above the sand surface	Inhibited the cellular development of aerenchyma	Shimamura et al., 2014
*Solanum dulcamara*	100 μM and 1 mM ABA	10- to 12-week-old plants were submerged up to 15 cm above the soil in glass containers	ABA arrested the AR development resulting in increased sensitivity	Dawood et al., 2016
*G. max*	5, 10, and 50 μM ABA	Two-day-old plants were flooded, and at 4 cm of water above the quartz sand surface	Improved soybean survival compared with waterlogging treatment alone	Komatsu et al., 2013
*S. lycopersicum*	1 mM NPA	Four-week-old plants were flooded up to the first node above the cotyledons	Inhibited AR growth after flooding.	Vidoz et al., 2010
*C. sativus*	10 mg/L NAA or 10 μM NPA	3- –week-stage seedlings water levels were raised 2 cm above the soil surface for 7 days	NPA compromised the of AR growth	Qi et al., 2020
*G. max*	50 μM JA	Seedlings were transferred to glass tube and covered it with plastic cap for waterlogging stress	Promoted plant growth under waterlogging by inducing stress-related proteins	Kamal and Komatsu, 2016
*C. sativus*	2.1 nM EBR	Waterlogging stress along EBR application was given to plants for 7 days consecutively before collecting samples	Increased the ethylene and production and homeostasis under waterlogging stress in plant roots	Ma and Guo, 2014
*Malus baccata*	50, 100, 200, 400, and 600 μM MT	After the seedlings developed to have four leaves, treated with MT by spraying or irrigation	Plant possessed better aerobic respiration to avoid waterlogging-induced damage	Zheng et al., 2017
*Medicago sativa*	100 μM MT	Foliar spray of MT was performed 1 day after waterlogging stress. Six- to seven-leaf stages were exposed to waterlogging by maintaining 1 cm above the soil surface and last for 10 days	Improved the production of endogenous melatonin and production of AR	Zhang R. et al., 2019

### Ethylene

Ethylene is a gaseous hormone in plants, and its diffusion rate is extremely low in water. The rapid accumulation of ET is an important way in which plants respond to waterlogging (Alpuerto et al., 2016; Hartman et al., 2019a,b).

1-Aminocyclopropane-1-carboxylic acid (ACC), the direct precursor in the biosynthesis of ET, is produced in large quantities under the catalysis of ACC synthase (ACS), and the process can occur under hypoxic conditions. ACC is converted to ET under the catalysis of ACC oxidase (ACO), but the process needs the participation of O_2_; thus, ACC needs to be continuously transferred from the hypoxic environment of the root system to the lower region of the plant’s aerobic part where the oxidation reaction can take place, finally producing ET. Rauf et al. (2013) found that waterlogging directly or indirectly activates the expression of *ACO5* and *ACS* genes in *Arabidopsis* and increases ET biosynthesis.

ET synthesis and perception are necessary for AR formation. Waterlogging treatment was carried out on 4-week-old tomato (*Solanum lycopersicum*) seedlings, and 500 μM aminoethoxyvinylglycine (AVG), which inhibits ET biosynthesis, was sprayed on the above-ground parts daily. After 72 h of waterlogging, AR primordia became visible in the stem base of waterlogged plants that had not been treated with AVG, and these primordia elongated and generated a large number of ARs within 7 days. The number of ARs in tomato plants treated with the inhibitor AVG was significantly lower than in untreated plants (Vidoz et al., 2010). Qi et al. (2019) found that treating cucumber seedlings with 1 mg/L 1-methylcyclopropene (1-MCP, an ET receptor inhibitor) before waterlogging inhibited the formation of ARs, whereas exogenous 10 μM ACC promoted the formation of ARs under waterlogging. Kim et al. (2018) not only significantly induced the occurrence of ARs on soybean plants but also increased the root surface area by exogenous application of 50, 100, or 200 μM ethephon, a synthetic plant growth regulator that produces ET when metabolized.

The production of endogenous ET is closely related to the development of aerenchyma cells (Kreuzwieser and Rennenberg, 2014; Mignolli et al., 2020). The expression of *ACS1* and *ACO5* is induced in rice root aerenchyma under hypoxia and promotes ET synthesis. At the same time, ET induces cortical cell death, mediated by ROS, leading to aerenchyma formation (Yamauchi et al., 2017). ET accumulates in roots under waterlogging as its biosynthesis continues and the diffusion rate in water is low. ET stimulates programmed cell death that occurs during the formation of lysogenic aerenchyma (Sasidharan and Voesenek, 2015). The accumulation of ET triggers the formation of lysosomal aerenchyma in maize (Yamauchi et al., 2016), rice (Yamauchi et al., 2017), and wheat (Yamauchi et al., 2014).

ET response factor (*ERF*) is an important transcription factor involved in plant responses to several different biotic and abiotic stresses. The *ERF* family genes specifically induce genes containing AGCCGCC elements and DRE/CRT *cis*-elements, activating or inhibiting the expression of downstream functional genes, and thereby mediating plant tolerance to various stresses (Yin et al., 2019). *ERF* transcription factors are regulated by ET, and exogenous ET significantly promotes *ERF* transcription in *Arabidopsis* and soybean (Hess et al., 2011; Tamang et al., 2014). ET regulates the response of *Arabidopsis* to hypoxia stress through *ERF73/HRE1* (Hess et al., 2011).

Group VII ET-response factors (*ERF*-VIIs) play an important role in ET signal transduction and plant responses to waterlogging (Gasch et al., 2016; Giuntoli and Perata, 2018). The gene *ZmEREB180*, a member of the *ERF*-VII family in maize, positively regulates the growth and development of ARs and the level of ROS: overexpression of *ZmEREB180* in maize also improves the survival rate after long-term waterlogging stress (Yu et al., 2019). The *PhERF2* protein binds directly with the promoter of ADH-related gene *ADH1-2. PhERF2*-RNAi lines had a mortality rate of 96% after flooding. Almost all *PhERF2-*overexpressing lines survived and showed faster and stronger recovery than WT plants (Yin et al., 2019).

The rice *Sub1A* (*Submergence1A*) gene is another member of the ET-response factor *ERF*-VII family. Overexpression of *Sub1A* enhanced the transcription of *ADH1* in transgenic rice and at the same time led to the enhanced ability to withstand waterlogging stress. Therefore, *Sub1A* gene could be the main determinant of submergence tolerance (Xu et al., 2006). Interestingly, the other two *ERF*-VII gene family members in rice, *SK1/2* (*SNORKEL1/2*), can also regulate the waterlogging tolerance of rice. Overexpression of *SK1/2* led to internode elongation and significantly improved the waterlogging tolerance of deepwater rice cultivars (Hattori et al., 2009).

However, *Sub1A* and *SK1/2* have opposite functions in regulating rice growth in response to waterlogging. *Sub1A* negatively regulates the GA response by limiting the degradation of the DELLA family protein SLR1 (Slender Rice-1), which are GA signal inhibitors, and thereby inhibits the elongation of rice (Fukao and Bailey-Serres, 2008; Hattori et al., 2009). Plants temporarily inhibit energy metabolism until water recedes, an effective long-term submergence strategy that occurs in rice mainly in non-deepwater cultivars (Xu et al., 2006; Bailey-Serres and Voesenek, 2008). In contrast, *SK1/2* stimulates GA synthesis, which promotes rapid growth of rice stems and internode petioles, a structural change that allows rice leaves to respire normally once they have extended away from the water. The reason was found to be that submersion induced ET accumulation in rice and positively regulated the stability of *OsEIL1a*, an ET-responsive transcription factor (Kuroha et al., 2018). OsEIL1a protein promotes SK1/2 transcription by directly binding to the SK1/2 promoter, and then SK1/2 mediates the expression of downstream genes to initiate shoot elongation (Kuroha et al., 2018). OsEIL1a also directly binds and transcribes the promoter of GA biosynthesis gene *SEMIDWARF1* (*SD1*), and SD1 protein promotes the synthesis of GA, mainly GA_4_, thus promoting shoot elongation (Kuroha et al., 2018). This evidence indicates that the *OsEIL1a–SD1–SK1/2* cascade is closely related to waterlogging tolerance in deepwater rice cultivars.

### Abscisic Acid

Abscisic acid (ABA) has a main role in regulating stomata by adjusting the size of guard cells, thereby regulating the water potential in plants. Because of this, ABA is considered to be a key hormone in water stress responses (Zhu, 2016; He et al., 2018).

Abscisic acid is involved in the development of root aerenchyma under waterlogging. The ABA concentration in soybean hypocotyls decreased rapidly under waterlogging, falling by 50% within 24 h compared with plants without waterlogging. In addition, secondary aeration tissues appeared after 72 h. Exogenous 1 μM ABA treatment inhibited the cell development of aerenchyma, suggesting that the formation of secondary aerenchyma required a reduction in the concentration of the negative regulatory factor ABA (Shimamura et al., 2014).

During waterlogging stress of *Solanum dulcamara*, following the rapid down-regulation of ABA biosynthesis and up-regulation of ABA decomposition, the ABA concentration in the stem and AR primordia decreased sharply (Dawood et al., 2016). Waterlogging resulted in ET accumulation in the lower stem and subsequently reduced ABA concentrations in the stem and AR primordia. 1 mM ABA treatment significantly inhibited the formation of ARs induced by waterlogging, whereas 100 μM ABA inhibitor (Fluridone) induced AR production (Dawood et al., 2016). These results showed that ABA, in contrast to ET, negatively regulated the formation of ARs under waterlogging. Kim et al. (2015) determined the plant hormone content in soybeans after waterlogging for 5 and 10 days and found that the ABA content significantly decreased. The ABA content in waterlogging-resistant lines was significantly lower than that in sensitive lines, indicating that ABA might be negatively correlated with waterlogging tolerance. Waterlogging increased shoot elongation in deepwater rice cultivars partly by reducing the endogenous ABA content and thereby increasing the GA concentration (Yang and Choi, 2006). Similarly, ET and its precursor ACC rapidly induced the expression of *OsABA8ox1*. In addition, ET receptor inhibitor 1-MCP pretreatment partially inhibited the expression of *OsABA8ox1*. These results indicated that the rapid decline of ABA in deepwater rice cultivars under waterlogging was partly controlled by ET-induced *OsABA8ox1* expression (Saika et al., 2007).

The relative expression of the kiwifruit (*Actinidia deliciosa*) gene *AdPDC1* encoding pyruvate decarboxylase was significantly up-regulated under waterlogging, suggesting that the gene played an important role in the waterlogging response. ABA down-regulated the expression of *AdPDC1* under waterlogging, whereas the overexpression of *AdPDC1* in *Arabidopsis* inhibited seed germination and root elongation under ABA treatment, indicating that ABA might negatively regulate *AdPDC1* under waterlogging (Zhang J.Y. et al., 2016).

However, other studies have shown that accumulation of ABA accelerated in the above-ground parts of the plant under waterlogging. ABA increased the accumulation of H_2_O_2_ and promoted stomatal closure, thus reducing the water loss from transpiration and improving the resistance of plants to waterlogging and related adverse environmental conditions. Overexpression of *AP2/ERF* family gene *RAP2.6L* in *Arabidopsis* promoted the expression of ABA biosynthesis genes, thus increasing ABA concentration. The increased ABA in *RAP2.6L*-overexpressing plants led to initiation of the antioxidant defense system and stomatal closure and finally resulted in reduced oxidative damage, delayed senescence, and significantly improved waterlogging tolerance (Liu et al., 2012). A significant increase in ABA content induced by waterlogging has been reported in cotton (Zhang Y. et al., 2016), wheat (Nan et al., 2002), and other crops. Komatsu et al. (2013) found that addition of 5, 10, and 50 μM ABA during waterlogging significantly improved soybean survival compared with waterlogging treatment alone. Similarly, pretreatment with 10 μM ABA had recorded affirmative responses in rice net assimilation rate, relative growth rate, and chlorophyll content under submergence (Saha et al., 2021).

### Auxin

Auxin (IAA) plays an important role in plant growth and development (Kazan and Manners, 2009; Lv et al., 2019). ET production, as an early response to waterlogging, can promote the transport of auxin, and conversely, the accumulation of auxin can prompt ET biosynthesis, further stimulating auxin transport to flooded parts of the plant, where the accumulation of auxin can induce ARs by initiating cell division. Exogenous application of the auxin transport inhibitor 1-naphthylphthalamic acid (NPA) to tomato (Vidoz et al., 2010), cucumber (Qi et al., 2019), and tobacco (McDonald and Visser, 2003) led to inhibition of AR growth after flooding.

The dynamic transport of auxin in plants is mediated by the auxin polar transport carrier protein PIN (PIN-FORMED), and treatment of rice with the transport inhibitor NPA decreased the expression of *OsPIN2*, suggesting that NPA might inhibit the production of ARs through an effect on PIN (Lin and Sauter, 2019). Similarly, when auxin polar transport was blocked in PIN expression–deficient mutants of *S. dulcamara*, the formation of ARs was inhibited, which further confirmed that AR production required auxin transport (Dawood et al., 2016).

However, some studies found that waterlogging reduced the content of IAA in soybean plants. Shimamura et al. (2016) found that the hypocotyl could form ARs and aerenchyma after 72 h of waterlogging, but physiological tests showed no significant difference within 72 h in the endogenous IAA concentration in the hypocotyl between the waterlogged and the control groups. This result showed that the accumulation of IAA was not a necessary condition for the formation of secondary aerenchyma in soybean hypocotyls under waterlogging.

Waterlogging can cause a large amount of carbohydrate consumption in plants, leading to energy shortage. Qi et al. (2020) first proposed a model for the interaction of sugars with auxin-induced AR initiation and elongation in waterlogged cucumber. Under waterlogging stress and in light conditions, photosynthesis supported the biosynthesis of sugars, whose accumulation induced auxin transport and subsequent signal transduction, and finally induced the formation of ARs in the hypocotyl.

### Gibberellin

GAs are one of the essential plant hormones regulating growth and development. GAs regulate multiple processes in plant growth and development, mainly by controlling the size and number of cells (Nelissen et al., 2012).

Studies on different genotypes of soybean found that GA content in waterlogging-tolerant lines significantly increased under waterlogging, and GA content in waterlogging-resistant lines was significantly higher than that in waterlogging-sensitive lines (Kim et al., 2015). Huang et al. (2018) determined physiological indexes of peanuts (*Arachis hypogaea*) under waterlogging and found that spraying GA on the leaf surface could promote the growth of upper and underground parts of peanut plants and significantly increase the yield. Wang et al. (2016) showed that exogenous GA could effectively reduce the MDA content in the leaves and roots of rape under waterlogged conditions, thus improving the tolerance of plants to waterlogging.

Treatment with inhibitors of GA biosynthesis significantly reduced internode elongation in rice under waterlogging (Hattori et al., 2009; Ayano et al., 2014). Mutations in GA biosynthesis (*Os1*, *OsCPS2*, *OsKS2*, *OsKS5*, *OsKO2*, *OsKAO*, *Os13ox*, *OsGA20ox1*, *OsGA20ox2*, *OsGA20ox3*, *OsGA3ox1*, *OsGA3ox2*) and signal transduction genes (*OsGID1*, *OsGID2*, *OsSPY*, *OsSEC*, *OsGAMYB*) also inhibited internode elongation (Ayano et al., 2014). Waterlogged rice plants treated with exogenous GA were able to restore internode elongation, enabling the leaves to respire normally once away from the flood water. GA has been shown to be a key hormone in improving rice tolerance under waterlogged conditions. Under waterlogging, GA participates in the *SK1/2* gene-mediated response pathway, and the GA content is up-regulated, leading to internode elongation. This structural change causes rice to extend above the water surface and reestablish gas exchange between plant tissue and the air (Hattori et al., 2009; Ayano et al., 2014). GA biosynthesis gene *SD1* was shown to be the cause of internode elongation under waterlogging. When submerged, the *SD1* gene was activated by *OsEIL1a*, an ET-responsive transcription factor, and SD1 protein promoted the synthesis of GA, mainly GA_4_, which promoted the rapid growth of leaf stalk internodes in rice (Kuroha et al., 2018). The results indicated that GA is centrally involved in promoting internode elongation in rice under waterlogged conditions.

### Salicylic Acid

Salicylic acid (SA) is a common phenolic compound in plants, which regulates the antioxidant mechanism of cells by inducing the expression of stress-related genes, thus enhancing the adaptability of plants to adverse conditions (Zhou et al., 2009; Hayat et al., 2010; Arif et al., 2020).

Salicylic acid, as a signal substance, can induce changes in physiological characteristics of waterlogged plants. Peach trees (*Prunus persica* L.) were subjected to waterlogging stress. Spraying exogenous SA on day 1 of waterlogging can significantly increase the activities of ethanol dehydrogenase, protective enzymes such as POD and CAT, and the content of proline in leaves and roots, thereby protecting leaves and root membranes from damage and stabilizing photosynthetic capacity of leaves as well as root activity. Together, these protective effects are conducive to the alleviation of waterlogging-induced stress (Wang et al., 2015).

An increase of SA content might be an important factor in tolerance of waterlogging stress. Studies have shown that SA regulates two different physiological responses. First, an increase in intracellular SA triggers a programmed cell death response, leading to an increase in lipid peroxidation in the root cell walls, which in turn leads to the development of aerenchyma cells within the root. Aerenchyma cells can increase oxygen transfer into the root tissues and alleviate waterlogging stress. Second, the accumulation of SA stimulates the formation of AR primordia and further enhances waterlogging tolerance by inducing the development of a large number of ARs (Kim et al., 2015).

Kim et al. (2015) measured SA content in soybean after 5 and 10 days of waterlogging and found that the content of SA in waterlogging-tolerant soybean PI408105A was significantly higher than that in the unstressed control, whereas the content of SA in waterlogging-sensitive soybean S99-2281 was not significantly different from that in the control. Elevated SA would stimulate the formation of ARs, promote gas exchange, and ultimately enhance waterlogging tolerance. Bai et al. (2009) found that spraying exogenous SA alleviated oxidative stress damage caused by hypoxia stress on plants, and enhanced the hypoxia tolerance of *Begonia occidentalis*. The above results indicate that appropriate SA level can promote the formation of ARs and aerenchyma, which is positively correlated with the waterlogging tolerance of plants.

### Jasmonic Acid

Jasmonic acid (JA) is a basic plant growth regulator that is known to be involved in the defense response produced by abiotic stress, but there are few studies on the relationship between JA and waterlogging tolerance (Per et al., 2018; Farhangi-Abriz and Ghassemi-Golezani, 2019; Raza et al., 2020; Wang J. et al., 2020).

Xu et al. (2016) found that the JA content in hypocotyl of Pepino, a waterlogging-sensitive cucumber line, was about twice that of the unstressed control after 2 days of waterlogging. However, JA content in the hypocotyl of the waterlogging-resistant line Zaoer-N decreased significantly during waterlogging to only 0.33 that of the control. The result suggested that JA is negatively correlated with the waterlogging tolerance of plants. However, other research showed that JA treatment inhibited root growth and the action of SA under waterlogging. Compared with the control, 649 different proteins were found in waterlogged soybeans treated with JA, which were mainly related to the stress response metabolite pathway, glycolysis, ethanol fermentation, and cell wall and cell tissue metabolism. The application of JA significantly reduced the damage to soybean plants under waterlogging and promoted plant growth by changing the proteomic profile (Kamal and Komatsu, 2016). There can be significant differences in JA content in different tissues of the same plant under waterlogging conditions. Under waterlogging stress, the JA content in citrus leaves increased significantly compared with the unstressed control, but the JA concentration in the root system decreased sharply. This might be caused by inhibition of the key lipoxygenase of the JA synthesis pathway under hypoxic conditions (Arbona and Gómez-Cadenas, 2008).

The interaction between JA and ET plays an important role in the formation and development of the root system and aerenchyma under waterlogging stress. Spraying methyl jasmonate on the leaves increased the content of ET (Hudgins and Franceschi, 2004). Thus, exogenous JA can increase the content of ET, which is beneficial in relieving waterlogging stress.

### Brassinosteroid

Brassinosteroid (BR) is a naturally occurring steroid in plants. BR can induce resistance to a variety of biological and abiotic stresses, thus promoting plant growth and development (Bajguz and Hayat, 2009; Huang et al., 2020; Nazir et al., 2021).

Exogenous 24-epi-brassinolide (EBR) promotes the transfer of carbohydrates from leaves to roots of cucumber seedlings under hypoxic stress, enhances the activity of glycolytic enzymes in the roots, and triggers the antioxidant enzymes activity and reduced ROS production, thus improving the resistance of the seedlings to hypoxic stress (Kang et al., 2009). EBR also improved enzyme activity related to cell wall degradation by promoting ET production in cucumber seedlings. It further promoted the expansion and loosening of cucumber hypocotyl and formation of ARs, thus improving the oxygen supply status of the plant and enhancing the tolerance of the plant to hypoxic stress (Ma and Guo, 2014).

The *Sub1A* gene, an ET-response factor *ERF-VII* family member, has different regulatory effects on brassinolide biosynthetic gene expression and rice shoot elongation under waterlogging, compared with exogenous BR. Exogenous BR pretreatment can activate the tolerance mechanism in waterlogging-tolerant rice genotypes and inhibit shoot elongation under waterlogging. Compared to the LOES, higher expression of BR biosynthesis genes was observed in the Sub1A rice genotype, which triggered the endogenous BR level. The enhanced BR level induced the transcription of GA catabolism gene *GA2ox7* resulting in reduced GA contents. At the same time, GA-mediated responses can be negatively regulated under submerged conditions by a DELLA family member, the GA-signal inhibitory factor SLR1 protein, so that the elongation of rice plants is inhibited (Schmitz et al., 2013). Therefore, BR limited shoot elongation by inhibiting GA biosynthesis and decreasing the action of GA in the rice *Sub1A* genotype.

Some selected recent studies on Phytohormones and plant growth regulator mediated waterlogging tolerance in plants are presented in Table 1.

### Melatonin

Melatonin (MT) is a phytohormone and an excellent antioxidant molecule that augments plant growth under adverse conditions (Sharif et al., 2018). The MT has been previously reported for its mitigatory role of numerous abiotic stresses (Sharif et al., 2018). Owing to that, research related to MT and its involvement in improving waterlogging stress tolerance is relatively less, and only few research articles are available (Moustafa-Farag et al., 2020).

The very first report over MT in response to waterlogging stress unraveled that it can mend plant tolerance by inducing the activity of antioxidant enzymes, suppression of harmful ROS, and maintained proper growth to ensure good yield (Chen et al., 2015). Following that, the young apple seedlings subjected to waterlogging stress were treated with MT (Zheng et al., 2017). The study showed that seedling treated with MT presented enhanced tolerance to waterlogging stress by triggering the generation of antioxidant enzymes activities, improved aerobic respiration, and photosynthetic machinery (Zheng et al., 2017). On the other hand, the application of MT significantly inhibited the deleterious effects of anaerobic respiration and MDA- and ROS-induced chlorosis (Zheng et al., 2017). The induced expression level of MT biosynthesis genes such as *MbT5H1*, *MbAANAT3*, and *MbASMT9* increased the production of endogenous MT in the seedlings treated with MT (Zheng et al., 2017). Therefore, it can be assumed that MT plays a key role in regulating the response of plants to waterlogging stress. The growth of the alfalfa plant has been hampered by the waterlogging stress by dysfunctioning the photosynthetic ability and boosted the generation of electrolyte leakage and MDA contents (Zhang Q. et al., 2019). The up-regulated expression level of PA biosynthesis genes also highlighted their involvement in regulating the alfalfa response to waterlogging stress (Zhang Q. et al., 2019). The application of MT at the rate of 100 μM over 6-week-old alfalfa seedlings displayed tolerance to waterlogging stress (Zhang Q. et al., 2019). The enhanced tolerance post–MT application was associated with the further induction in the expression of PAs biosynthesis genes (*SPDS*, *SPMS*, and *ADC*). Also, the exogenous MT treatment not only increased the endogenous MT level but also stabilized the normal functioning of other biochemical and physiological parameters (Zhang Q. et al., 2019). Further, the MT-treated plants under waterlogging stress exhibited decreased expression pattern of ET biosynthesis and signaling genes (*ACS*, *ACO*, and *ERF*) (Zhang Q. et al., 2019). This means that MT and ET possess an antagonistic relationship under waterlogging stress. However, no report is available to confirm the antagonistic crosstalk between MT and ET. *P. persica* is considered one of the most hypoxia-intolerant stone fruits. However, waterlogging, which causes hypoxia, occurs frequently in southern China, where peaches are commercially important (Gu et al., 2020). The application of MT at the rate of 200 μM substantially augmented the antioxidant activities, suppressed the lipid peroxidation, and H_2_O_2_, positively regulated the size of aerenchyma for better anaerobic respiration activities and induced mRNA level of Ca^2+^ signaling and hypoxia-related ERF VII transcription factor genes (Gu et al., 2020). Therefore, it can be suggested that the application of MT positively regulates the ET homeostasis, which is an important and crucial factor in inducing waterlogging stress tolerance.

## Inducing Waterlogging Tolerance *via* Genetic Engineering

The manipulation of targeted plant genes to increase the production capacity or tolerance against a certain stress is becoming the need of the day (Lemay and Moineau, 2020). As the climate threat looms over the safe production of agronomic and horticultural crops, genome editing techniques can play a significant role in decreasing the adverse environmental effects (Lemay and Moineau, 2020). Previous studies have shown that the deleterious effects of the waterlogging stress can be mimicked by utilizing the genome editing tools. For example, the overexpression of *AtACO5* gene in *Arabidopsis* triggered the ET production, and cell expansion activities resulted in enhanced tolerance against waterlogging stress (Rauf et al., 2013). The overexpression of *CsARN6.1* gene in cucumber facilitates the formation of ARs independent of hormonal generations. However, the increased number of ARs in the overexpressed *CsARN6.1* lines was associated with the intense cellular activities and hydrolysis of the ATP energy packets (Xu et al., 2018). The ERF transcription factors are directly involved in the regulation of waterlogging stress. A member of ERF transcription factor family *PhERF2* was characterized in *petunia* (Yin et al., 2019). Up-regulation in the transcript abundance of *PhERF2* was observed under waterlogging stress. To further highlight the role, the *PhERF2* overexpressed lines were generated, which displayed enhanced tolerance to waterlogging stress. On the contrary, the RNAi line of *PhERF2* showed sensitivity to waterlogging (Yin et al., 2019). The genes influence the alcoholic fermentation process such as *ADH1-1*, *ADH1-2*, *ADH1-3*, *PDC1*, and *PDC2* induced and suppressed in overexpressed and silenced plants, respectively (Yin et al., 2019). Similarly, the induced expression levels of *NtPDC*, *NtADH*, *NtHB1*, *NtHB2*, *NtPCO1*, and *NtPCO2* genes in *AdRAP2.3* overexpressed tobacco plants presented its association with the enhanced waterlogging stress tolerance (Pan et al., 2019). In wheat, waterlogging stress can significantly hinder the physiological activities particularly photosynthesis, which ultimately reduce the grain yield and affect overall yield. The constitutive expression of *TaERFVII.1* gene in wheat alleviated the negative effects of waterlogging stress by boosting the immunity resulting in increased grain weight per plant, improved survival rate, and better chlorophyll content of leaves (Wei et al., 2019). On the other hand, the compromised expression of *TaERFVII.1* in silenced plants also decreased the transcript of several waterlogging−responsive genes (Wei et al., 2019). Interestingly, the constitutive expression of *TaERFVII.1* did not negatively impact both plant development and grain yield under standard conditions by suppressing the *TaSAB18.1* gene (Wei et al., 2019). The barley *HvERF2.11* when overexpressed in *Arabidopsis* triggered the expression level of antioxidant enzyme biosynthesis genes (*AtSOD1*, *AtPOD1*) and ET biosynthesis gene (*AtACO1*), conferring resistance to waterlogging stress (Luan et al., 2020). The HD-ZIP I subfamily gene *HaHB11* was overexpressed in the *Arabidopsis* and exposed to waterlogging stress (Cabello et al., 2016). The transgenic *Arabidopsis* plants carrying gain-of-function *HaHB11* gene not only induced the tolerance to waterlogging stress but also increased the biomass and yielded more seeds than control by inducing the glucose and sucrose level (Cabello et al., 2016). In addition, the *HaHB11* were notably involved in the increment of expression of genes involved in the alcohol fermentation (Cabello et al., 2016). Multiple studies highlighting the importance of genetic engineering in augmenting the immunity of plants to waterlogging stress are presented in Table 2.

**TABLE 2:** Listed studies related to improved waterlogging tolerance *via* genetic engineering.

Plant species	Gene	Ways	Functional response	References
*Arabidopsis*	*AtACO5* and *AtACS*	Overexpression	Overexpression of *ACO5* and *ACS* genes enhanced the generation of ethylene under waterlogging	Rauf et al., 2013
*Arabidopsis*	*AtLDH*	Overexpression	Overexpression of *LDH* significantly enhanced the PDC activity and hypoxia resistance	Dolferus et al., 2008
*Arabidopsis*	*AtRAP2.6L*	Overexpression	Overexpression of *RAP2.6L* promoted the expression of ABA biosynthesis genes, antioxidant defense system, and stomatal closure	Liu et al., 2012
*Oryza sativa*	*RBOHH*	Knockout by CRISPR/Cas9	Knockout of the *RBOHH* gene in rice reduces both ROS accumulation and aerenchyma formation	Yamauchi et al., 2017
*O. sativa*	*OsSK1* and *OsSK2*	Overexpression	Overexpression of *SK1/2* led to internode elongation	Hattori et al., 2009
*O. sativa*	*OsSub1A*	Overexpression	Overexpression of *Sub1A* enhanced the transcription of *ADH1* in transgenic rice and led to the enhanced ability to withstand waterlogging stress	Xu et al., 2006; Fukao and Bailey-Serres, 2008; Hattori et al., 2009
Maize	*ZmEREB180*	Overexpression	Overexpression of *ZmEREB180* in maize improves the survival rate after long-term waterlogging stress *via* induced AR formation	Yu et al., 2019
*Petunia*	*PhERF2*	Overexpression and RNAi	*PhERF2*-RNAi lines had a mortality rate of 96% after flooding. *PhERF2-*overexpressing lines survived and showed faster and stronger recovery than WT plants.	Yin et al., 2019
Kiwi fruit	*AdRAP2.3*	Overexpression	Overexpression of *AdRAP2.3* in tobacco improved the PDC and ADH enzyme activities and improved the expression levels of waterlogging mark genes in roots	Pan et al., 2019
Wheat	*TaERFVII.1*	Overexpression and VIGS	The constitutive expression of *TaERFVII.1* gene in wheat boosted the immunity and overall yield. The silenced plants also decreased the transcript of several waterlogging−responsive genes	Wei et al., 2019
Barley	*HvERF2.11*	Overexpression	*HvERF2.11* overexpression triggered the expression level of antioxidant enzyme biosynthesis genes and ethylene biosynthesis gene in *Arabidopsis*	Luan et al., 2020
Sunflower	*HaHB11*	Overexpression	Overexpressed in the *Arabidopsis* simultaneously impacted tolerance and biomass in a positive manner	Cabello et al., 2016

## Conclusion and Outlook

The regulation of plant growth and development processes under waterlogging stress is very complex, with different crops, different varieties of the same crop, and different growth periods of the same crop often showing great differences, while different plant species evolved different adaptation strategies. At present, research on crop waterlogging tolerance is mainly carried out from the perspective of morphological, structural, physiological, biochemical, and metabolic gene signal regulation. The most effective ways to enhance plant waterlogging tolerance will be (1) improving cultivation management to reduce the direct damage to crops caused by waterlogging and (2) using modern molecular biology technology to discover the key genes regulating waterlogging tolerance and verify their functions.

Building on existing research results and aiming to address identified problems, the following aspects should receive increased attention in future research on plant waterlogging tolerance:

(1) Current studies focus mainly on the vegetative growth stage of plants under waterlogging stress. However, the molecular responses during seed germination, early seedling morphogenesis, and late reproductive growth under waterlogging stress are neglected topics that warrant further study.
(2) Although a large number of related genes regulating plant waterlogging tolerance have been obtained by transcriptomics, proteomics, and other methods. However, most of them are preliminary study and required functional characterization.
(3) There is a need to exploit additional genetic resources for waterlogging tolerance, using both isolated populations and natural populations to identify waterlogging tolerance–related genes.
(4) The hormonal-induced waterlogging resistance has been studied extensively. Majority of the available studies mainly reported the effects of growth hormones on vegetative stages under waterlogging stress. Although studies are missing to investigate the role of these phytohormones when waterlogging stress happens at reproductive stages of the plant. Hormonal crosstalk under waterlogging stress in the early developmental stages of plant has been investigated and is presented in Figure 2. However, it could be interesting to examine the complex hormonal crosstalk under waterlogging stress during reproductive stages, such as how these hormones ensure plant productivity under prolonged waterlogging stress. Additionally, is there an unknown genetic factor(s) controlling phytohormone-mediated cascades under waterlogging condition? Therefore, it is of great importance to elucidate these mechanisms to develop waterlogging resilience plants to increase crop productivity particularly in the areas that have poor soil drainage properties, those affected by frequent heavy rainfall, and areas with duplex soil.

## Author Contributions

JP wrote the manuscript. RS, XX, and XC revised and finally approved the manuscript for publication. All the authors contributed to the article and approved the submitted version.

## Conflict of Interest

The authors declare that the research was conducted in the absence of any commercial or financial relationships that could be construed as a potential conflict of interest.
